# Editorial on special issue “The 11th International Conference on Surface Plasmon Photonics (SPP11)”

**DOI:** 10.1515/nanoph-2025-2000

**Published:** 2025-12-10

**Authors:** Takuo Tanaka, Wakana Kubo

**Affiliations:** Metaphotonics Research Team, RIKEN Center for Advanced Photonics, Wako, Saitama 351-0198, Japan; Department of Electrical Engineering and Computer Science, Tokyo University of Agriculture and Technology, Koganei, Tokyo 184-8588, Japan

**Figure 1:**
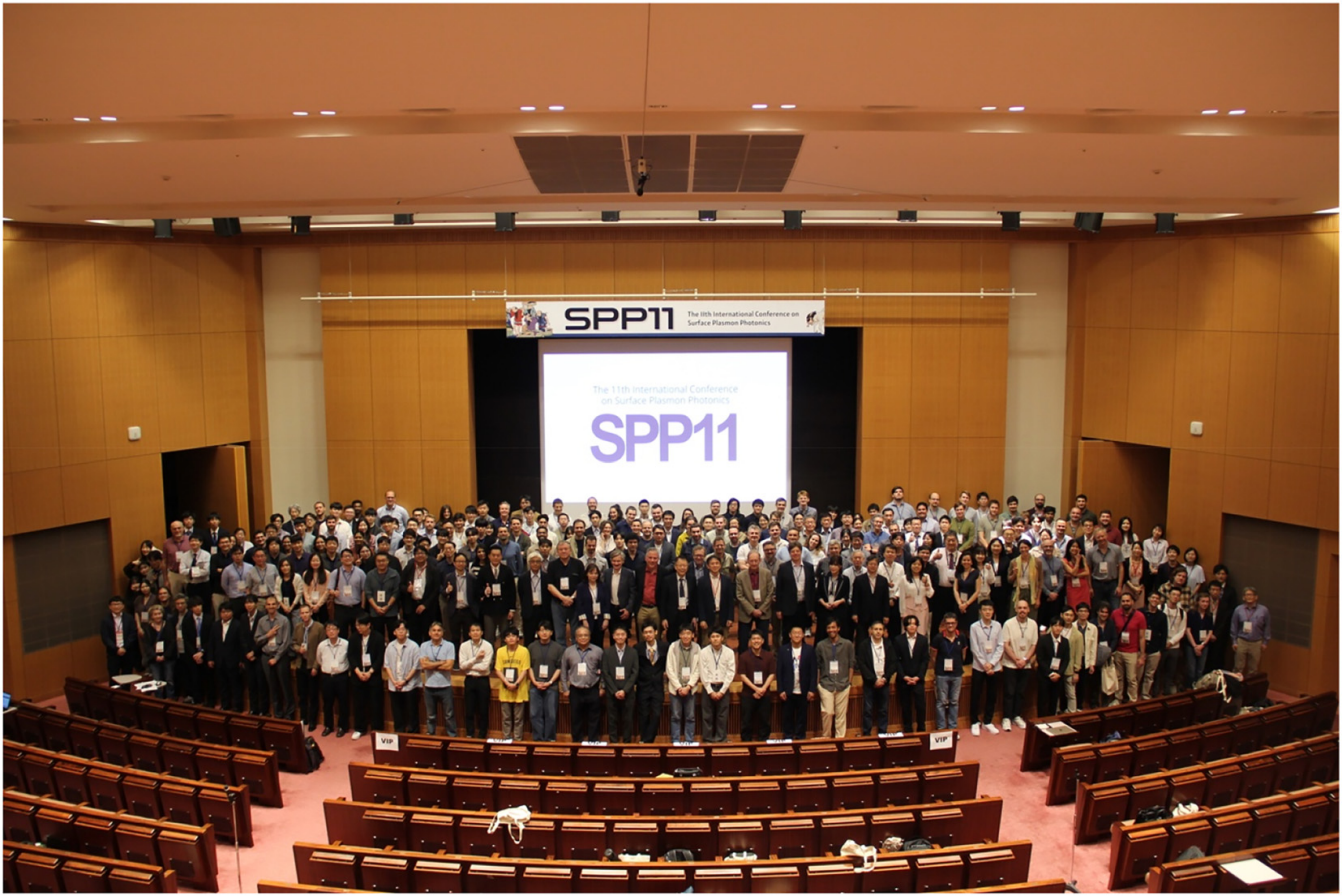
A group photograph of SPP11 on May 19th, 2025.

The 11th International Conference on Surface Plasmon Photonics (SPP11) was held at Hitotsubashi Hall, National Center of Sciences Building in Tokyo, Japan, from May 19th to 23rd, 2025 as Figure 1.

The International Conference on Surface Plasmon Photonics (SPP) is a biennial independent and non-profit conference series widely regarded as the premier series in the field of plasmonics, metamaterials, and nanophotonics. Previous venues of the conference series include: Obernai, France (SPP0, 2001), Granada, Spain (SPP1, 2003), Graz, Austria (SPP2, 2005), Dijon, France (SPP3, 2007), Amsterdam, The Netherlands (SPP4, 2009), Busan, South Korea (SPP5, 2011), Ottawa, Canada (SPP6, 2013), Jerusalem, Israel (SPP7, 2015), Taipei, Taiwan (SPP8, 2017), Copenhagen, Denmark (SPP9, 2019), and Houston, USA (SPP10, 2023).

Plasmonics has a long history, and fundamental research has been extensively conducted across a wide range of topics. Similarly, nearly three decades have passed since the first proposal and realization of metamaterials. Today, plasmonics and metamaterials are no longer pursued only as subjects of fundamental science or as “technologies of the future”; they have evolved into practical technologies for which concrete applications are being widely investigated, optimized, and implemented.

Participants shared their latest research findings and discussed future directions in the rapidly evolving field of plasmonics, nanophotonics, and metamaterials. The conference covered both the fundamental principles and emerging applications of plasmonics, nanophotonics, and metamaterials, featuring keynote and invited presentations from internationally recognized leaders, along with contributed oral and poster sessions.

This special issue of *Nanophotonics* brings together selected contributions from SPP11 and related research presented at the conference. It highlights the latest advances in plasmonics, nanophotonics, and metamaterials, reflecting not only the remarkable scientific and technological progress achieved by the community, but also the expanding frontiers and interdisciplinary opportunities that continue to shape this dynamic field.

J. Ahn et al. [1] contribute the memorial paper for Professor Dai-Sik Kim, who was general chair of SPP5 and passed away on November 11th, 2024, and reviews the scientific contributions and vision of Prof. Kim in near-field optics, plasmonics, and THz spectroscopy. Authors also highlight major breakthroughs and their impact through personal accounts from collaborators. Xie et al. [2] reveal hidden meso-chirality in plasmonic nanoparticles emerging from cancellation between absorption- and scattering-based chiral responses. Authors further show that such concealed chirality can be strong and broadband in realistic nanostructures. An et al. [3] show that adjusting the wavelength and polarization of excitation light allows controlled generation of plasmonic topological quasiparticles in a metasurface. Authors also visualize their distinct near-field textures, confirming tunable skyrmion and meron formation. Chaves et al. [4] present a hydrodynamic model that captures nonlocal magnetoplasmon behavior in anisotropic 2D materials. Authors further show how nonlocal and quantum effects reshape their dispersion and optical conductivity. Biehs and Bondarev [5] identify topological singularities in transdimensional plasmonic films that strongly influence their reflection coefficients. Authors further show that these features can induce unusually large Goos–Hänchen shifts in the visible range. Engelberg et al. [6] develop a nature-inspired design strategy enabling wide-FOV achromatic metalenses. Authors demonstrate its effectiveness through a metalens achieving ±20° FOV and broadband operation. Li et al. [7] report nanofocusing of vortex beams using a hyperbolic-metamaterial hyper-grating platform. Authors also predict the formation of deeply subwavelength optical skyrmions through numerical simulations. Liu et al. [8] achieve strong coupling between a split-ring resonator and an ITO ENZ mode to enhance second-harmonic generation. Authors additionally demonstrate more than three-order-of-magnitude improvement compared to conventional Au/ITO structures. Chen et al. [9] introduce a far-field super-resolution imaging method that visualizes resonance-mode distributions in semiconductor nanowires by exploiting photothermal nonlinear scattering. Authors further show that extracting higher-order nonlinear components via differential SAX microscopy enables resolving sub-diffraction periodic modes that remain hidden in conventional linear imaging. Dayi et al. [10] report stable and anisotropic second-harmonic generation from monocrystalline copper microflakes synthesized using an oxidation-resistant on-substrate growth method. Authors further reveal a strong cross-polarized SHG response with C_3v_ symmetry and demonstrates that the SHG signal remains stable for hours under continuous femtosecond excitation, confirming copper’s robustness as a nonlinear plasmonic platform. Xie et al. [11] present a reconfigurable metasurface platform that achieves six fully independent phase-modulation channels by jointly tuning incident polarization and phase-change material crystallinity. Authors further validate this dual-state framework through dynamically switchable multifocal metalenses and multichannel holography, and even exploits inter-state crosstalk for progressive optical information encoding. Chu et al. [12] introduce a metasurface-based Fourier ptychographic microscopy platform that combines a compact 4-f metalens system with a programmable TFT illumination panel to achieve wide-field, high-resolution quantitative phase imaging without mechanical scanning. Authors further show that integrating a residual convolutional neural network enables single-shot high-resolution phase reconstruction, achieving nearly twofold resolution enhancement and accurate dry-mass quantification of biological cells. Tai et al. [13] present a dual-band spectral filter array integrated with a telecentric lens to achieve real-time surface plasmon resonance imaging using a monochrome camera. Authors further show that γ-based spectral contrast analysis, enabled by pixel-shift demosaicing, improves the refractive-index detection limit by nearly two orders of magnitude compared with wavelength-shift methods. Uenoyama et al. [14] present an analytical model that visualizes angle-resolved carrier generation and collection pathways in silicon photodetectors enabled by plasmonic diffraction from a gold nanograting. Authors further show that the model closely matches experiments measuring photocurrents in both illuminated and non-illuminated adjacent pixels, revealing how diffraction-guided light propagation enhances NIR detection efficiency. Gao et al. [15] present flexible TiO_2_ nanoantenna stickers that directionally enhance photoluminescence from YAG:Ce phosphor plates by tuning lattice geometry, plate thickness, and distributed Bragg reflector (DBR) layers. Authors further show that these detachable metasurface stickers achieve up to eleven-fold enhancement with improved forward directionality and color tuning, offering a versatile approach for efficient, compact illumination control. Řepa et al. [16] investigate whether plasmonic antennas with a large cross-section can function as charge reservoirs capable of enhancing near-field response under plane-wave excitation. Authors reveal through combined EELS measurements and electromagnetic simulations that increased antenna volume instead leads to dominant radiative losses, suppressing field enhancement and disproving the charge-reservoir hypothesis. Endo et al. [17] demonstrate cavity-mediated coupling between a local cyclotron resonance mode and finite-momentum magnetoplasmon modes in a Landau polariton system by using slot cavities on a GaAs two-dimensional electron gas and terahertz time-domain magnetospectroscopy. Authors further show that the observed upper-polariton splitting is accurately captured by a multimode Hopfield model and finite-element simulations, establishing a platform for multimode light–matter interactions linking zero- and finite-momentum excitations in the ultrastrong-coupling regime. Chu et al. [18] present a polarization-encoded color image platform based on refractory HfN plasmonic metasurfaces, where orthogonally oriented nanoantenna arrays support visible LSPRs that generate distinct color channels under x- and y-polarized illumination. Authors further show that these metasurfaces maintain their plasmonic response and dual-QR-code encryption functionality even after 900 °C annealing, enabling thermally robust, CMOS-compatible optical security and anticounterfeiting applications. Omori and Iwami [19] propose a wavelength- and angle-multiplexed metasurface hologram that uses a single, polarization-independent SiN cross-shaped meta-atom to independently control the phase of three primary RGB wavelengths without sacrificing pixel density. Authors further demonstrate high-definition, crosstalk-free full-color 3D image reconstruction by spatially dividing the target images and precisely superimposing them via angle correction based on Rodrigues’ rotation formula, overcoming fundamental limitations of previous full-color metasurface holography.

We, conference organizers, believe that this special issue provides a comprehensive overview of recent research activities by leading scientists in the field of plasmonics, metamaterials and nanophotonics. Finally, we sincerely appreciate all contributions from the authors to this special issue.
